# Absence of IL-1β positively affects neurological outcome, lesion development and axonal plasticity after spinal cord injury

**DOI:** 10.1186/1742-2094-10-6

**Published:** 2013-01-14

**Authors:** Francesco Boato, Karen Rosenberger, Sofie Nelissen, Lies Geboes, Eva M Peters, Robert Nitsch, Sven Hendrix

**Affiliations:** 1 Department of Morphology & BIOMED Institute, Campus Diepenbeek, Hasselt University, Agoralaan Gebouw C, Diepenbeek, BE 3590, Belgium; 2 Present Address: Université Pierre et Marie Curie, Institut de la Vision, 17 rue Moreau, Paris, 75012, France; 3 Department of Neurology, Charité Universitätsmedizin, Charitéplatz 1, Berlin, D-10117, Germany; 4 Psychoneuroimmunology, University-Medicine Charité, Charité Center 12 for Internal Medicine and Dermatology, Berlin, D-10117, Germany; 5 Department of Psychosomatic Medicine, Justus-Liebig-University, Klinikstrasse 32, D-35392, Gießen, Germany; 6 Institute of Microanatomy and Neurobiology, University Medical Center, Johannes Gutenberg University, Langenbeckstrasse 1, 55131, Mainz, Germany

**Keywords:** IL-1β, Corticospinal tract, Glial scar, Spinal cord compression injury

## Abstract

Precise crosstalk between the nervous and immune systems is important for neuroprotection and axon plasticity after injury. Recently, we demonstrated that IL-1β acts as a potent inducer of neurite outgrowth from organotypic brain slices *in vitro*, suggesting a potential function of IL-1β in axonal plasticity. Here, we have investigated the effects of IL-1β on axon plasticity during glial scar formation and on functional recovery in a mouse model of spinal cord compression injury (SCI). We used an IL-1β deficiency model (IL-1βKO mice) and administered recombinant IL-1β. In contrast to our hypothesis, the histological analysis revealed a significantly increased lesion width and a reduced number of corticospinal tract fibers caudal to the lesion center after local application of recombinant IL-1β. Consistently, the treatment significantly worsened the neurological outcome after SCI in mice compared with PBS controls. In contrast, the absence of IL-1β in IL-1βKO mice significantly improved recovery from SCI compared with wildtype mice. Histological analysis revealed a smaller lesion size, reduced lesion width and greatly decreased astrogliosis in the white matter, while the number of corticospinal tract fibers increased significantly 5 mm caudal to the lesion in IL-1βKO mice relative to controls. Our study for the first time characterizes the detrimental effects of IL-1β not only on lesion development (in terms of size and glia activation), but also on the plasticity of central nervous system axons after injury.

## Introduction

IL-1β is a 17 kDa protein and is one of the most extensively studied proinflammatory cytokines. IL-1β is almost undetectable in the undamaged central nervous system (CNS), but its expression increases several fold after injury (including neurotoxic stimuli, ischemia and trauma) [1]. Microglia are the principal cells expressing IL-1β, but many other resident cells (including astrocytes and neurons) or invading cells are also able to produce the cytokine [2]. IL-1β mainly acts by activating the immune response, fostering the production of inflammatory mediators and other cytokines. Interestingly, when applied on healthy neurons, IL-1β does not cause damage or death; however, it induces a number of cellular reactions such as changes in intracellular calcium concentrations and ionic conductances [3,4]. In an organotypic model we recently demonstrated that IL-1β is a potent inducer of neurite outgrowth from brain slices [5], suggesting a potential function of IL-1β in axonal plasticity. This is in contrast to its well-studied contribution to both acute neuronal loss and chronic neurodegeneration [1]. Despite the intensive research on IL-1β in recent decades, its role in the CNS remains far from fully understood. The *in vitro* effects of IL-1β are very heterogeneous. IL-1β supports survival of dorsal root ganglion neurons [6] and pyramidal neurons *in vitro*[7], but it also induces depolarization, increases the spike frequency and enhances vulnerability of hippocampal neurons induced by N-methyl-d-aspartate receptor-mediated increase of intracellular calcium [4]. IL-1β also has strong effects on astrocytes, promoting activation, proliferation and production of neurotoxic mediators, as well as survival promoting factors [8]. Studies from our group showed either no effects of IL-1β on neurotrophin-induced outgrowth from dorsal root ganglion neurons [9] and spinal cord explants or a beneficial effect on axonal growth from brain slices *in vitro*[5]. The *in vivo* role of IL-1β is also not clear. Intracerebroventricular administration of IL-1β during ischemic damage after permanent middle cerebral artery occlusion results in a highly enhanced infarct volume (~92%) [10], similar to the effect of systemic [11] or local [12] administration of the cytokine in mice with middle cerebral artery occlusion. Moreover, IL-1β leads to exacerbated cell death and neurodegeneration in other experimental models of CNS trauma [13,14]. In contrast, there is evidence that IL-1β is necessary for proper remyelination of the CNS following death of mature oligodendrocytes since IL-1β^−/−^ nerve fibers are unable to remyelinate properly after cuprizone-induced demyelination [15]. Finally, IL-1β contributes to sensory nerve regeneration *in vivo* following sciatic nerve injury [16,17].

In this study, we have investigated the effects of increased local levels of IL-1β compared with IL-1β absence (in IL-1βKO mice) after compression of the spinal cord [18]. In contrast to our *in vitro*-based hypothesis, but in line with the results on IL-1βKO mice reported in a recently published study from the Shioda laboratory [19], we here demonstrate for the first time detrimental effects of IL-1β on lesion development, in terms of lesion size and glial activation, and on the plasticity of CNS axons *in vivo* after injury.

## Materials and methods

### Spinal cord compression injury

All experiments with C57BL/6 wildtype (WT) mice and homozygous mice deficient in IL-1β [20] (IL-1βKO) (females, 8 to 12 weeks old) were performed in accordance with the German guidelines on the use of laboratory animals. Spinal cord injury, corticospinal tract (CST) tracing and subsequent analysis were carried out following a standardized protocol [18,21]. Briefly, C57BL/6 mice and IL-1β-deficient mice underwent a dorsal laminectomy at thoracic level T8, and the compression of the spinal cord was induced with a modified SPI Correx Tension/Compression Gage (Penn Tool, Maplewood, NJ, USA) at 10 cN for 3 seconds. For recombinant IL-1β (rIL-1β) and PBS application, a piece of Gelfoam (Pharmacia & Upjohn, Erlangen, Germany) soaked in 5 μl solution with PBS alone or with 1 or 20 μg rIL-1β was placed directly on top of the injured spinal cord and in contact with the perforated dura before suturing the muscles. Important to note in these experiments is that when recombinant cytokine was applied, a Gelfoam patch was in direct contact with the injured spinal cord, and this led to a lower score in control mice compared with the WT mice in the knockout experiments. The rIL-1β *in vivo* dosage was based on *in vitro* results coming from our group [5] demonstrating that rIL-1β increases axonal outgrowth when applied in a high therapeutic dosage in a well-established organotypic slice culture model [22-25]. The effective dosage in that study (500 ng rIL-1β in 500 μl medium) was substantially higher than the concentrations found *in vivo* after spinal cord injury (300 pg/ml in spinal cord (1 cm) homogenate 6 hours after injury). In the first experiment we therefore applied a high therapeutic dosage of 20 μg rIL-1β in Gelfoam, also taking into account that the dispersion of the cytokine is higher *in vivo* than *in vitro*, that the time of observation is longer (2 days for *in vitro* experiments and 14 days for *in vivo* experiments) and that the lesion volume in the spinal cord is much bigger than a 350 μm thick slice of the enthorinal cortex. Furthermore, to distinguish between local and systemic effects on functional recovery, a 100 μl solution of PBS alone or with 1 μg rIL-1β was also applied systemically by intraperitoneal injection immediately after injury.

### Behavioral analysis

The spinal cord compression injury (SCI) mice were tested over 14 days for functional recovery with the Basso Mouse Scale (BMS) [26], which is a locomotor rating scale ranging from 0 to 9 (0 = complete hind limb paralysis; 9 = normal locomotion). In BMS testing, mice are scored according to the mobility of the hind limbs for a period of 4 minutes in an open field by two investigators carefully blinded to experimental groups. Furthermore, since subscores for each parameter of the BMS can be used to measure individual locomotor features [26] and since correct foot placing correlates with proper CST function [27,28], stepping performance and correct paw positioning were evaluated as previously described [18]. The analysis of the stepping emphasized whether plantar stepping was present in <50% or in >50% of the steps (scores 0 and 1, respectively). For the scoring of paw positioning, we assessed whether the paws were rotated at both initial contact and lift-off (score 0), parallel at initial contact but rotated at lift-off (score 1), or parallel at both initial contact and lift-off (score 2). For both stepping performance and paw positioning, the score for each animal was then represented as a percentage, taking as 100% a score of, respectively, 1 and 2. For the BMS, stepping performance and paw positioning analysis we used the mean of the left and right hindlimb scores for each animal. Data shown represent mean values for all the animals of each experimental group ± standard error of the mean and were analyzed using a two-way analysis of variance as described previously [26].

### Corticospinal tract tracing and analysis

For biotinylated dextran amine (BDA, 10%; Invitrogen, Darmstadt, Germany) tracing, a small hole was drilled into the skull directly after SCI (after suturing the back muscles) and a Hamilton syringe was inserted into the motor cortex to apply 2 μl of 10% solution of the anterograde tracer. At the end of the observation period, CST fibers were visualized by diaminobenzidine staining on paraformaldehyde-fixed longitudinal cryosections (20 μm) of the spinal cord. BDA-labeled nerve fibers of the corticospinal tract were quantified at defined distances caudal to the lesion center in the complete microscopic field along the dorso-ventral axis at a total magnification of × 400. Counted fibers are shown as a percentage of the total number of labeled fibers within a standardized 20 μm wide area of the dorso-ventral diameter of the CST at level C4. Fibers were counted on five to seven adjacent sections with a clearly recognizable lesion and CST end (presenting retraction bulbs), and only the axons fulfilling the criteria outlined by Steward and colleagues [29] were included in the analysis. Data represent mean values ± standard error of the mean and were analyzed using the Mann–Whitney U test.

### Evaluation of lesion size, lesion width and degree of gliosis

Longitudinal spinal cord cryosections, 20 μm thick (obtained from transcardially perfused animals at the end of behavioral analysis period), were preincubated in PBS with 10% normal goat serum containing 0.5% Triton X-100 for 60 minutes at room temperature. Incubation with primary antibodies was carried out overnight at 4°C. Secondary antibodies were applied for 1 hour at room temperature. For measurement of the lesion size and gliosis, three to six sections per animal, the central part of the spinal cord included, were analyzed. The lesion size and lesion width were evaluated by staining against ionized calcium binding adaptor molecule 1 (Iba1) using rabbit polyclonal antibodies (Wako Chemicals, Neuss, Germany). Alexa Fluor 488 goat anti-rabbit antibodies (Invitrogen) were applied as secondary antibody. The Iba1-positive area was quantified using image analysis software (ImageJ open source software; National Institutes of Health, Bethesda, MD, USA) and averaged for each animal after the analysis of three to six sections. The lesion size was evaluated on sagittal sections and represents the mean area in the center of the spinal cord (corresponding to the lesion center, including the CST end), while the lesion width represents the total number of sagittal sections presenting the lesion, multiplied by the thickness of each section. Iba1 intensity was calculated for the same area and was found not to differ significantly between the experimental groups (data not shown). While the use of an Iba1-positive area to measure the lesion size is not a classical procedure, in this study we could not use traditional glial fibrillary acidic protein (GFAP) immunoreactivity measures for this goal (as often found in the literature and as performed in previous work from our laboratory [18]); this was due to the reduced GFAP immunoreactivity in the IL-1βKO mice, which did not allow for comprehensive analysis. Iba1 immunoreactivity in the lesion, however, correlated perfectly well with the lesion size found using bright-field microscopy, with the added advantage that Iba1 immunoreactivity was much easier to distinguish from the background. Astrogliosis was evaluated using staining against GFAP with mouse mAb (Sigma-Aldrich, St Louis, MO, USA). Alexa Fluor 568 goat anti-mouse (Invitrogen) was used as secondary antibody. Quantification of GFAP expression was performed as previously described [18,30] by means of intensity analysis using ImageJ software within an area 100 μm wide along the dorso-ventral axis of the spinal cord, extending from 600 μm cranial to 600 μm caudal to the lesion center. Additionally, the total intensity of GFAP staining in the white matter in the same area was calculated for each animal in the rostro-caudal axis. Data represent the mean ± standard error of the mean and were analyzed using the Mann–Whitney-U test.

## Results

**Figure 1:**
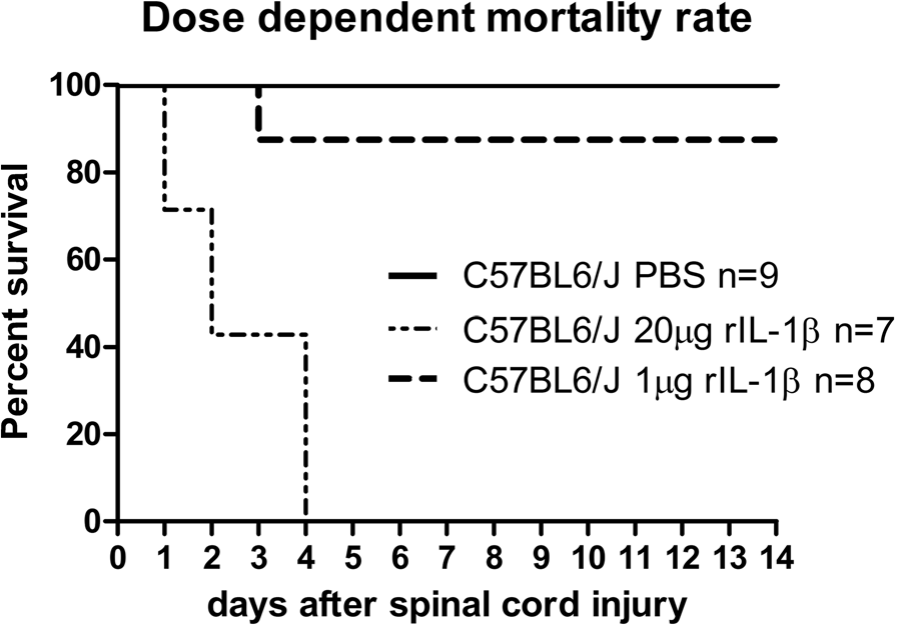
**Dose-dependent mortality after spinal cord compression injury. **The mortality rate in mice with local administration of recombinant IL-1β (rIL-1β) in Gelfoam directly after spinal cord compression injury (SCI) was 100% in mice treated with 20 μg rIL-1β, and was significantly higher compared with mice treated with 1 μg rIL-1β or only with PBS. *P* <0.0001 using the log-rank test.

**Figure 2:**
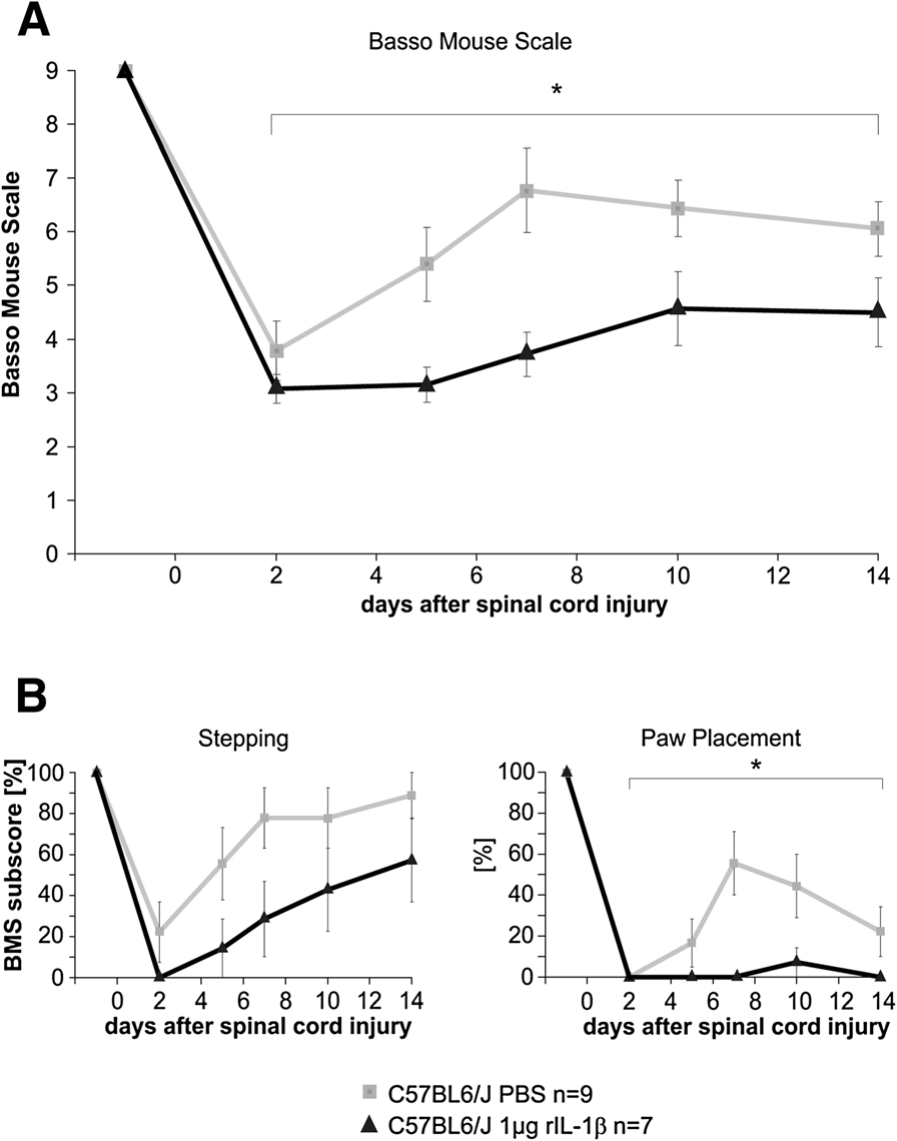
**Application of recombinant IL-1β impairs neurological outcome after spinal cord compression injury. **(**A**) Locomotion analysis using the Basso Mouse Scale (BMS) showed significant worsening of neurological outcome after spinal cord compression injury (SCI) and local administration of recombinant IL-1β (rIL-1β) in Gelfoam directly after SCI. The rIL-1β-treated mice scored more than 1 point of the BMS less than control mice (scoring respectively 4.5 and 6), most probably reflecting lack of coordination and consistent plantar stepping in the treated mice. (**B**) The paw positioning subscore differed significantly between the two groups, while the stepping subscore did not. *P <0.05, two-way analysis of variance.

**Figure 3:**
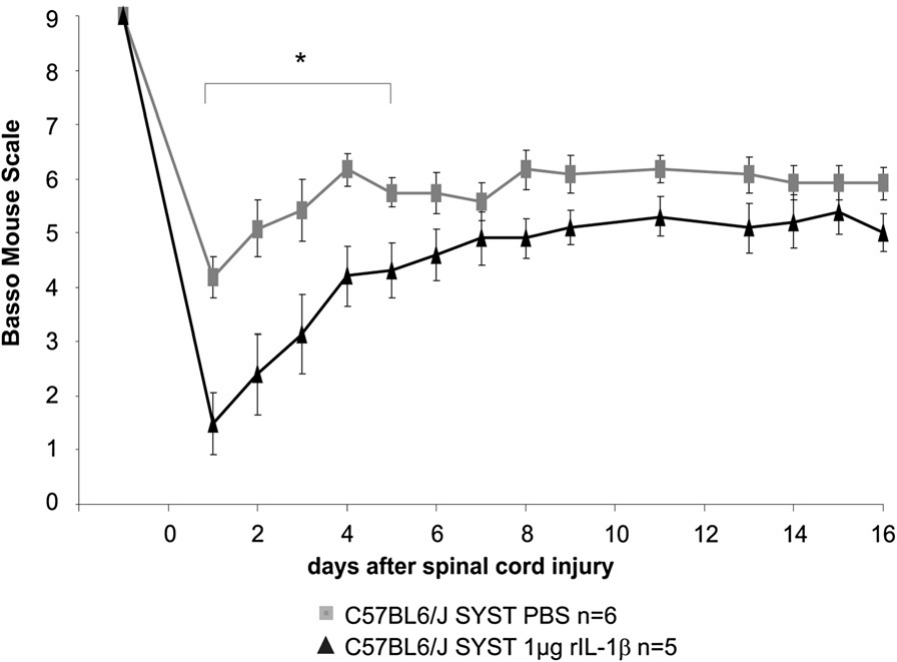
**Systemic application of recombinant IL-1β impairs neurological outcome after spinal cord compression injury. **Locomotion analysis using the Basso Mouse Scale (BMS) showed a significant early worsening of neurological outcome after spinal cord compression injury (SCI) and systemic administration of recombinant IL-1β (rIL-1β) compared with control mice. At days 1 and 2 after SCI, the rIL-1β-treated mice scored more than 2 points of the BMS lower than the control mice, but the significant difference was progressively lost and they scored in the same range of values as control mice at day 7 after lesion. *P <0.05, two-way analysis of variance.

In a first experimental approach we applied a high dosage of rIL-1β to mice that underwent spinal cord injury. To our knowledge this is the first study to investigate the *in vivo* effect of rIL-1β on axonal plasticity in the CNS, so the choice of the rIL-1β *in vivo* dosage was based on *in vitro* results from our group. Perilesional administration of 20 μg rIL-1β in Gelfoam immediately after SCI resulted in 100% mortality, with all mice (*n* = 7) dying within the first 4 days after the operation (Figure 1). We refrained from repeating the experiment to perform necropsies to diagnose the cause of death for obvious ethical reasons. Based on these results, we used a drastically reduced amount of cytokine (1 μg in 5 μl PBS), which substantially reduced the mortality (only one out of eight operated mice died 3 days after injury). The application of 1 μg rIL-1β significantly impaired functional performance compared with mice treated with PBS alone (Figure 2A). At the end of the investigation period (14 days after injury) the two experimental groups varied by more than one point on the BMS (from about 4.5 for rIL-1β-treated mice to 6 for PBS-treated mice), mainly reflecting a substantial difference in coordination and consistency of plantar stepping during walking. To rule out the possibility that the effect of rIL-1β-mediated worsened neurological outcome after SCI was due to a systemic effect of the cytokine, we applied 1 μg IL-1β systemically (intraperitoneal injection) after lesion (Figure 3). Under these conditions the treated mice showed a significant worsening of neurological outcome from day 2 to day 5 of the observation period, scoring more than 2 points of the BMS less than control mice in the first 2 days. They progressively improved at a higher rate if compared with mice treated only with PBS, and by day 7 of the scoring the significant difference between the two groups was lost. 

**Figure 4:**
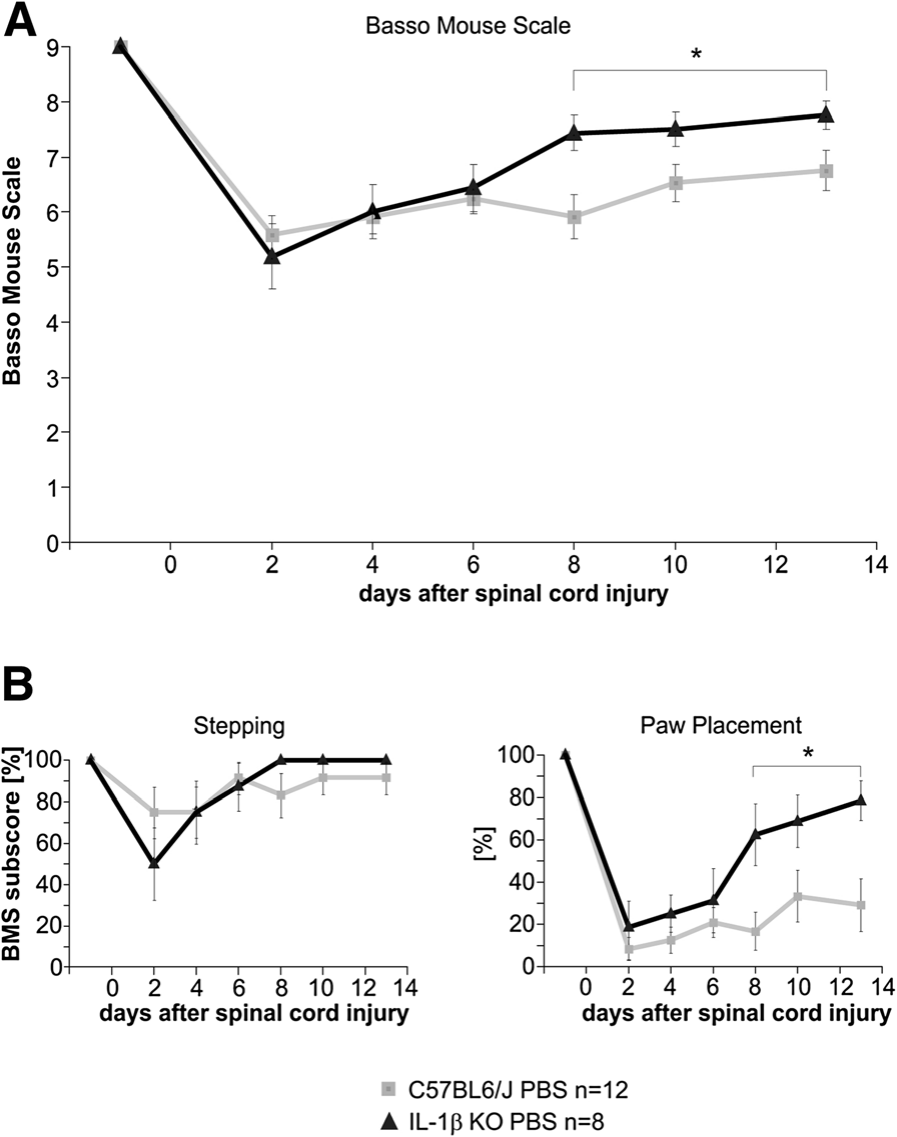
**Absence of IL-1β in IL-1βKO mice promotes functional outcome after spinal cord injury. **(**A**) Locomotion analysis using the Basso Mouse Scale (BMS) showed a significant increase of neurological outcome after spinal cord compression injury (SCI) in IL-1βKO mice, as evidenced by a difference of1 point of the BMS between treatment groups (6.8 for wildtype (WT) mice and 7.8 for IL-1βKO mice). (**B**) The paw positioning subscore was significantly different between the two groups. Conversely, the stepping subscore was almost identical. *P <0.05, two-way analysis of variance.

**Figure 5:**
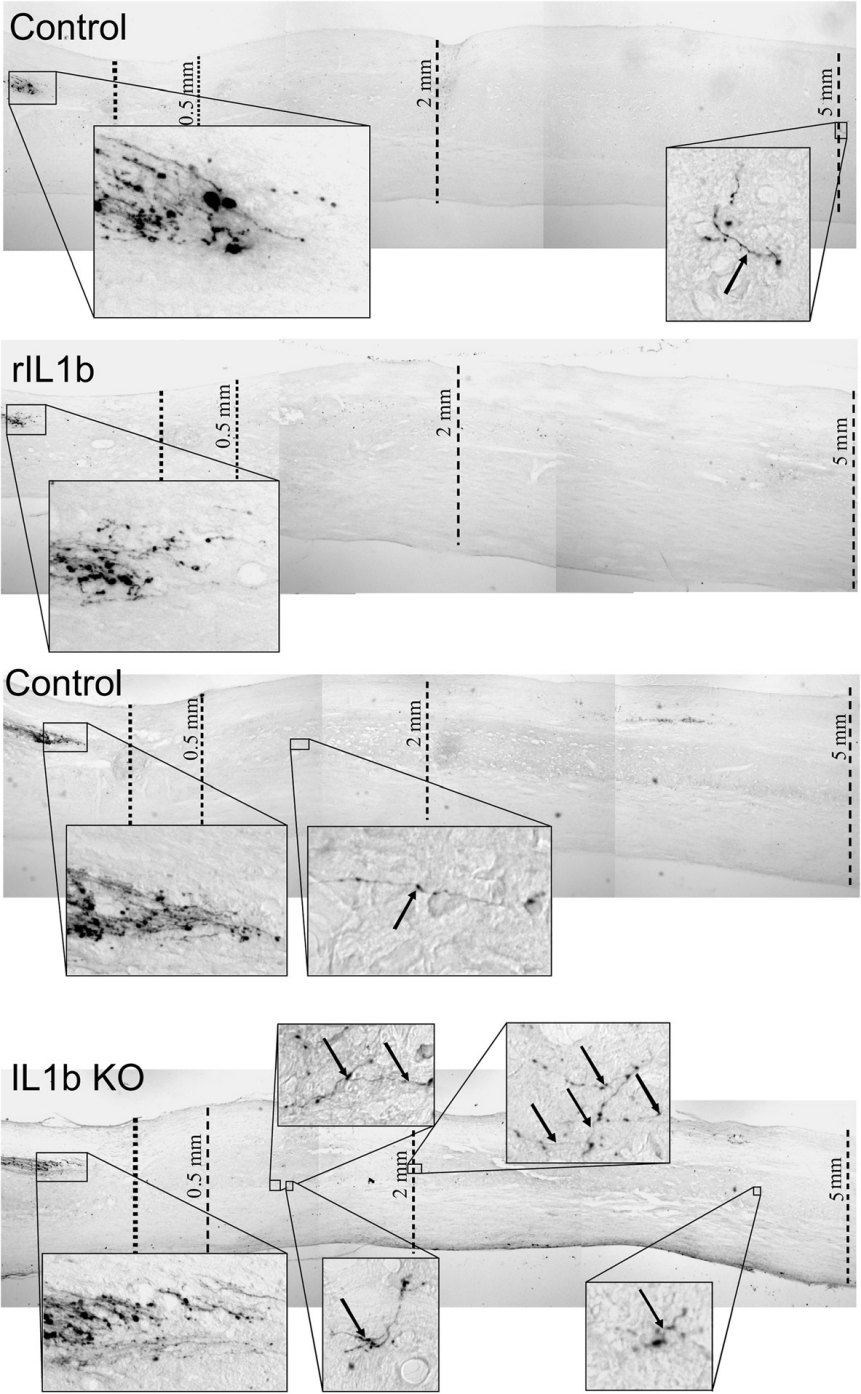
**Administration of recombinant IL-1β or absence of IL-1β alters numbers of corticospinal tract fibers. **(**A**), (**B**) Representative micrographs of the area of the spinal cord between the corticospinal tract (CST) end and 5 mm caudal to the lesion center (LC). Higher magnification panels highlight the area between the end of the CST and the LC and one selected area (recombinant IL-1β (rIL-1β)) to four selected areas (IL-1βKO) caudal to the LC, where diaminobenzidine-positive fibers could be detected. Arrows indicate CST fibers caudal to the LC.

**Figure 6:**
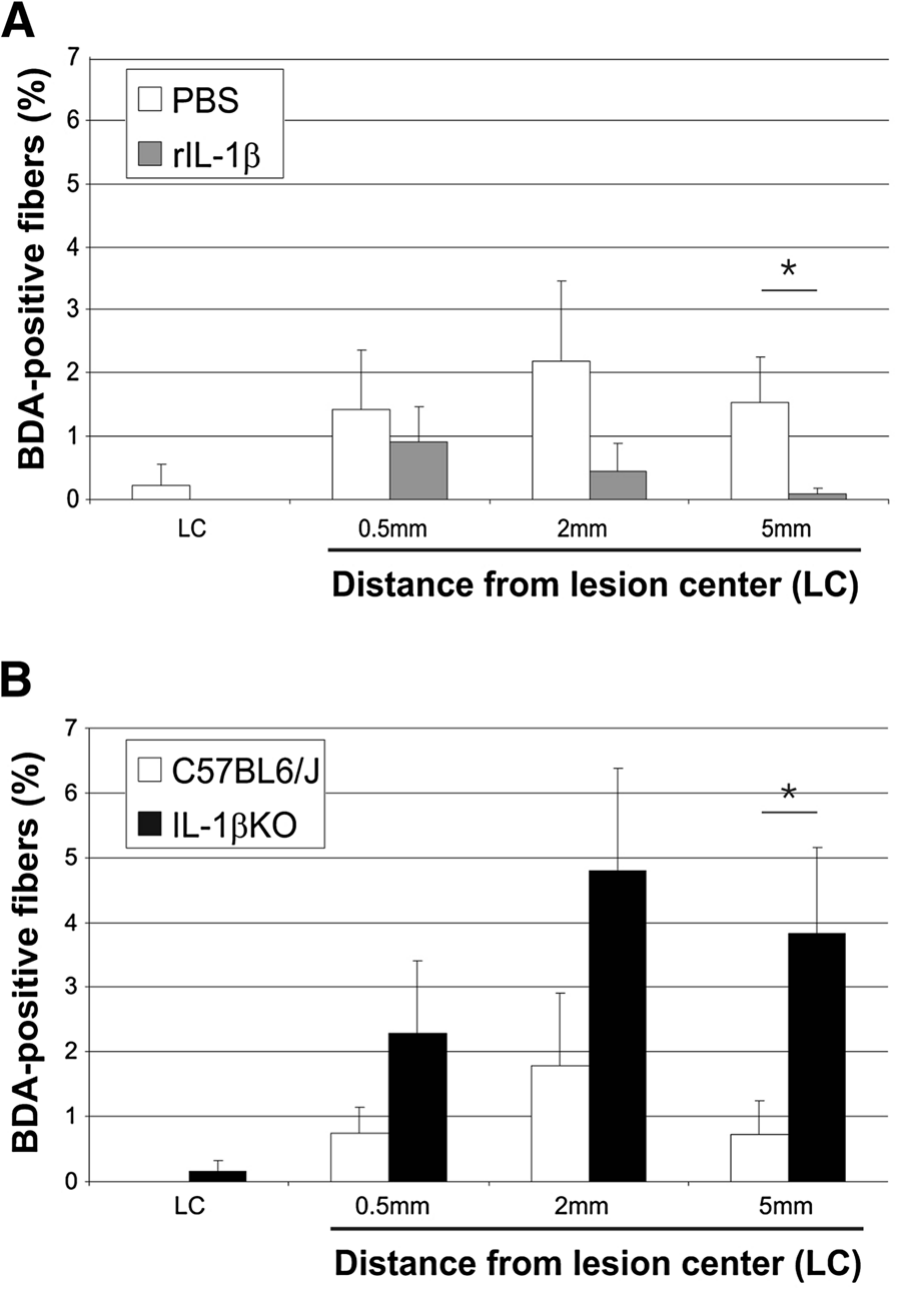
**Quantification of biotinylated dextran amine-positive corticospinal tract fibers in recombinant IL-1β-treated or IL-1βKO mice. **(**A**) The quantity of corticospinal tract (CST) fibers (shown as a percentage of the total number of biotinylated dextran amine (BDA)-positive fibers at C4 level in a standardized, 20 μm wide area [18]) was significantly decreased 5 mm caudal to the lesion in recombinant IL-1β (rIL-1β)-treated mice compared with controls. (**B**) Conversely, the percentage of CST fibers of IL-1βKO mice increased about fivefold compared with controls. Bars represent the percentage of CST fibers at the lesion center (LC) and in the area 0.5 mm, 2 mm and 5 mm distal to the LC. *P <0.05; *n* = 7 mice (PBS), *n* = 6 mice (rIL-1β), *n* = 9 mice (C57BL6/J), *n* = 6 mice (IL-1βKO). Values throughout are represented as mean ± standard error of the mean and *P* values were determined using the Mann–Whitney U-test.

**Figure 7:**
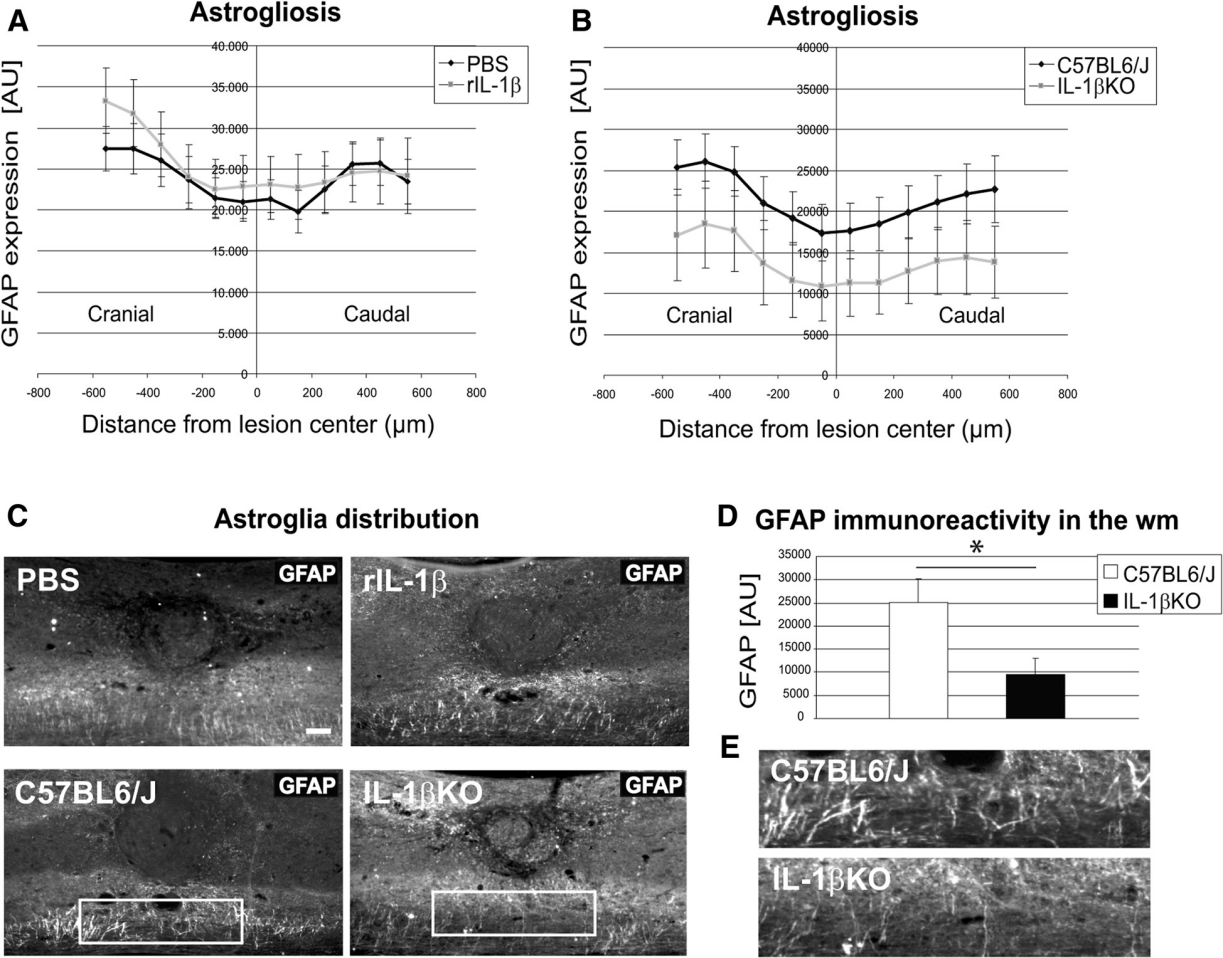
**Reduced glial fibrillary acidic protein expression in IL-1βKO white matter after spinal cord compression injury. **(**A**), (**B**) Quantification of the intensity of glial fibrillary acidic protein (GFAP) immunoreactivity in the entire dorso-ventral axis of the spinal cord from 600 μm cranial to 600 μm caudal to the lesion center (LC) in a standardized, 100 μm wide, area showed no significant difference in astrocytic reactions between PBS-treated and recombinant IL-1β (rIL-1β)-treated animals (**A**) or between wildtype controls and IL-1βKO animals (**B**). (**C**) Representative micrographs of spinal cord sections stained with GFAP showing the perilesional astroglia distribution for the four different study conditions. Upper panels: comparison of PBS-treated and rIL-1β-treated spinal cord. Lower panels: comparison of wildtype control with IL-1βKO spinal cord. (**D**) Quantification of GFAP intensity in a standardized area limited to the white matter (wm) shows a significant difference of more than 60% in immunoreactivity and astroglia expression when using IL-1βKO mice compared with controls. (**E**) Higher magnification of the boxes in (**C**) representing GFAP expression in the white matter of control and KO mice. *P <0.05; *n* = 5 mice (PBS), *n* = 5 mice (rIL-1β), *n* = 7 mice (C57BL6/J), *n* = 5 mice (IL-1βKO). Scale bar = 100 μm.

Conversely, the absence of IL-1β in IL-1βKO mice significantly promoted functional recovery after SCI compared with WT mice (Figure 4A), as indicated by a difference of 1 point of the BMS between experimental groups (from about 6.8 for the WT mice to about 7.8 for IL-1βKO mice). Analysis of the stepping subscore of the BMS showed a negative trend after administration of rIL-1β that did not reach significance, while in the paw positioning subscore a statistically significant difference was found (Figure 2B). On the contrary, IL-1β deficiency led to an almost identical stepping score but significantly improved the paw positioning score (Figure 4B), indicating that this parameter (degree of rotation of the hind paws) is of particular relevance to the difference in the functional recovery not only between PBS and rIL-1β-treated mice, but also between knockout and WT mice. In the next step, we analyzed the number of BDA-traced CST fibers as a marker for axonal plasticity induced by absence or increased levels of IL-1β at defined distances from the lesion center (Figures 5 and 6). Treatment of the injured mice with rIL-1β significantly reduced the number of labeled axons 5 mm distal to the lesion center compared with PBS-treated mice (Figure 5, upper panels and Figure 6A). In contrast, IL-1β absence in IL-1βKO mice led to a significant increase of BDA-positive fibers in the area 5 mm distal to the lesion and to the development of complex branches (Figure 5, lower panels and Figure 6B). Quantification of BDA-positive fibers, which were normalized (on spinal cord cross-sections of the spinal cord) to the total number of labeled CST fibers cranial to the lesion center, showed that the percentage of fibers present 5 mm distal to the lesion was reduced about 15-fold in rIL-1β-treated mice compared with PBS-treated mice (Figure 6A) and was increased about fivefold in IL-1βKO mice compared with WT (Figure 6B). So far, these data provided strong evidence for a neurodegenerative effect of rIL-1β applied on the site of the lesion after spinal cord injury, while genetic depletion of IL-1β resulted in a significantly improved recovery after injury and had a strong beneficial effect on plasticity (including local sprouting) of CST fibers. GFAP immunoreactivity did not significantly differ in mice with or without rIL-1β administration (Figure 7A, C upper panels) and was also not significantly different between WT and IL-1βKO mice when analyzing the entire dorso-ventral axis of the spinal cord (Figure 7B, 7C lower panels). In contrast, GFAP distribution was highly reduced (>60%) in IL-1βKO mice when focusing the analysis on the white matter (Figure 7D), as shown in a representative photomicrograph (Figure 7E) demonstrating a substantially reduced astrogliosis after SCI in the absence of systemic IL-1β. 

**Figure 8:**
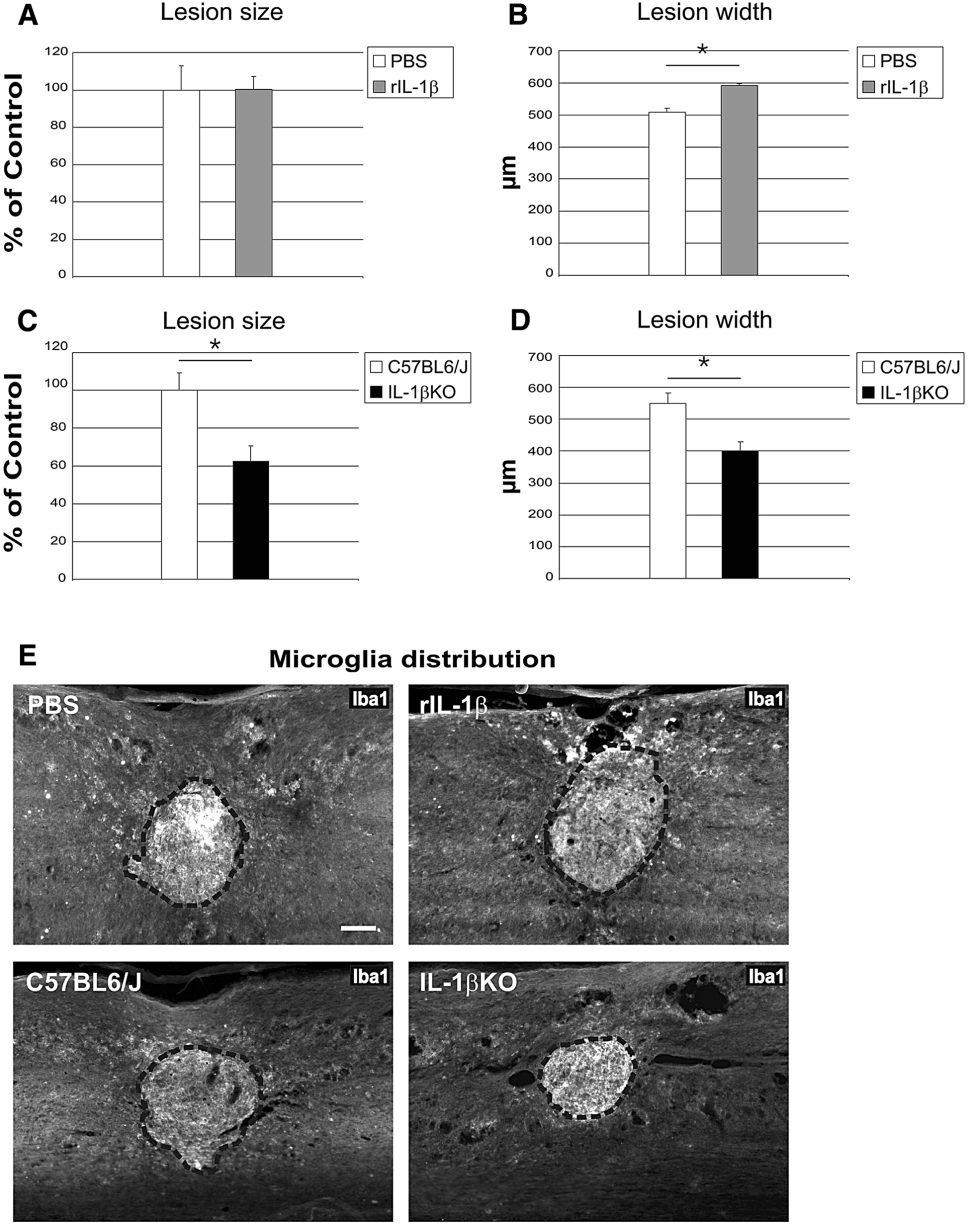
**Application of recombinant IL-1β and its deficiency influence lesion size after spinal cord compression injury. **(**A**) to (**D**) Quantification of the lesion size and lesion width based on a clearly distinguishable Iba1-positive area. Lesion size measurement in the central sections of recombinant IL-1β (rIL-1β)-treated mice indicated no difference compared with controls (**A**), while the lesion width was about 20% greater (**B**). Both the lesion size (**C**) and the lesion width (**D**) were reduced by 40% and 25%, respectively, in IL-1βKO mice compared with controls. (**E**) Representative micrographs of Iba1 immunoreactive microglia distribution around the compression injury site in spinal cord sections. Upper panels: comparison of PBS-treated and IL-1β-treated spinal cord. Lower panels: comparison of wildtype control with IL-1βKO spinal cord. Dashed line, area of the lesion. Iba1 intensity did not differ significantly between groups (data not shown). *P <0.05; *n* = 5 mice (PBS), *n* = 5 mice (rIL-1β), *n* = 7 mice (C57BL6/J), *n* = 5 mice (IL-1βKO). Scale bar = 100 μm.

The rostro-caudal lesion size (mean area on sagittal sections in the center of the lesion) and the lateral lesion width (corresponding to the number of sagittal sections that included the lesion, multiplied by the thickness of each section) were determined by assessing the clearly demarcated Iba1-positive area, but not the sparse Iba1 immunoreactivity present in the surrounding spared tissue (Figure 8). Application of rIL-1β did not influence the size of the lesion in the center of the spinal cord (Figure 8A), but did result in a lesion almost 100 μm wider compared with PBS-treated mice (from 500 μm in control mice to 600 μm in rIL-1β-treated mice; Figure 8B). On the contrary, systemic deficiency of IL-1β resulted in a significantly smaller lesion (almost 40% smaller than in WT mice; Figure 8C), as shown in a representative photomicrograph of the center of the lesion (Figure 8E lower panels); this was also reflected in a reduced lesion width (25% smaller in IL-1βKO mice; Figure 8D). 

## Discussion

In the present study we show for the first time that IL-1β exerts detrimental effects on the plasticity of CNS axons after SCI. These data are in striking contrast to our recent *in vitro* study, which revealed that IL-1β acts as a potent inducer of axon outgrowth from organotypic brain slices *in vitro.* IL-1β is one of the most extensively studied proinflammatory cytokines; however, controversial debate continues as to its function in the CNS. *In vivo*, IL-1β may exert detrimental effects on damaged nervous tissue [1], but some evidence exists for a beneficial impact on myelination [15] and on peripheral nerve regeneration following sciatic nerve injury [16,17]. After SCI, IL-1β and its receptor are upregulated in rodents and humans [13,30,31] in all resident cells of the CNS (including neurons, but mainly astrocytes and microglia), with a peak of mRNA expression 12 hours after lesion [31]. A functional consequence of increased IL-1β expression may be apoptosis induction, suggested by a threefold increase in caspase-3 activity that can be reversed by administration of the IL-1 receptor antagonist for 72 hours following lesion [32]. Direct application of IL-1β has also been shown to affect the behavioral outcome after glutamate-induced experimental spinal cord injury [33]. Even if the precise mechanism of action of IL-1β is still not perfectly clear, an extensive literature describes the pathways that are influenced by this cytokine. Of particular interest is the observation that IL-1β injections stimulate macrophage activation and myelin clearance in spinal cord white matter, while an absence leads to an increased number of intact myelin sheaths [34]. Furthermore, IL-1β application abrogates neurotrophin-induced neuronal cell survival *in vitro*[35,36]. Moreover, administration of IL-1β intrathecally activates p38 mitogen-activated protein kinase, and leads to high levels of inducible nitric oxide synthase and release of nitric oxide [37]. However, none of these studies investigated the influence of IL-1β on CNS plasticity. The present study demonstrates *in vivo* that the sum of all these negative effects in the CNS appears to abrogate potential IL-1-dependent axon elongation expected from our recent *in vitro* study [5].

Here, we provide the first *in vivo* evidence for a substantial IL-1β effect on plasticity and lesion development. We analyzed the effect of locally applied rIL-1β and its constitutive deficiency on functional recovery, CST fibers and astrogliosis in a mouse model of SCI, which mimics the most common type of spinal cord injury in humans [38]. Based on previous *in vitro* findings demonstrating that a high therapeutic dosage of rIL-1β increases axonal outgrowth in an organotypic slice culture model [5] we administered perilesionally a high dose of rIL-1β after SCI (20 μg rIL-1β in Gelfoam). Unfortunately, this local application was lethal. However, a lower, nonlethal dosage of rIL-1β (1 μg rIL-1β in Gelfoam) led to a significantly impaired functional recovery according to the BMS. We used a mild lesion to ensure that any possible negative effect of the application of the cytokine could be revealed. This treatment also resulted in a highly reduced number of BDA-positive CST fibers caudal to the lesion compared with PBS-treated mice. Consistently, the analysis of the injured spinal cords of IL-1β-deficient mice revealed a close to fivefold increase in the number of CST fibers caudal to the lesion compared with WT mice.

These mice also displayed a significantly improved neurological outcome. Based on morphological criteria [29], BDA-positive fibers counted caudal to the lesion appeared to present newly formed fibers, but due to the nature of the lesion we cannot exclude a small percentage of (undetected) sparing. Counted fibers could thus be a mixture of newly established fibers derived from the site of the lesion and of those sprouting from uninjured fibers.

It is reasonable to assume that multiple spinal motor systems are positively affected by the absence of IL-1β (and the consecutively reduced astrogliosis as discussed hereafter). The improvement in paw placement as an indicator of CST function [27,28] and increased numbers of anterogradely labeled CST nerve fibers in the IL-1β-deficient injured spinal cord support the concept that enhanced CST plasticity may significant contribute to the improved clinical outcome demonstrated in our study.

Substantial differences between the *in vivo* and *in vitro* model may explain why IL-1β stimulates neurite outgrowth *in vitro*[5] but has a negative impact on axon plasticity in the present *in vivo* study. The *in vitro* study was performed using organotypic slice cultures from postnatal brains as previously described [22-25]. Acute brain slices as used in our study, should be considered a model for the early, highly acute phase of CNS trauma since they are acutely excised from of the living brain; most neurons are axotomized, the blood–brain barrier is heavily damaged, high levels of neuronal death appear, and astrocytes as well as many immune cells are activated [39,40]. Contrastingly, in the *in vivo* model used here, the SCI is followed by at least three different inflammatory phases (acute, subacute and chronic) that are characterized by dramatic differences, for example, in terms of cytokine levels as well as immune cell activation and migration patterns [41]. The *in vivo* effects of IL-1β on functional parameters become clearly detectable about 2 weeks after the highly acute phase. IL-1β may therefore exert direct and/or indirect effects mainly in the subacute or early chronic phase. In a landmark paper, the Kapfhammer group demonstrated that neurite outgrowth can only be reliably studied in embryonic and postnatal brain slices until 4 days after birth [42]. Brain slice experiments are therefore performed with embryonic or early postnatal brains. In later developmental phases, neurite outgrowth from slices is substantially reduced or absent. A further difference between the models is the absence of systemic neuroendocrinological influences in the brain slice model. The contradicting results in our brain slice study *in vitro*[5] and the present *in vivo* study may thus be due to differences in the CNS region (cortex versus spinal cord), the inflammatory phase, the developmental stage (postnatal versus adult) or the presence or absence of systemic neuroendocrinological influences.

In the present study we also show significantly reduced astrogliosis in the perilesional white matter in IL-1βKO mice. This is in line with the role of IL-1β as an astroglial growth factor in the mammalian brain (promoting proliferation) [43] and with the IL-1β-dependent astrocyte activation following CNS injury, demonstrated in a murine corticectomy model [44], leading to exacerbated astrogliosis. However, in contrast to these studies, which describe early stages after brain injury, we here demonstrate that reduced astrogliosis in the absence of IL-1β is still present 2 weeks after injury. Consistently, we also find a greatly reduced lesion size (and consequently more spared spinal cord tissue).

While the results of the present study are encouraging to further investigate IL-1β modulation for spinal cord repair, they should be interpreted with care keeping in mind the complexity of the plethora of potential mechanisms as outlined in the introduction. Further studies are needed to elucidate the intricate network of IL-1β mechanisms of action after SCI, which is likely to be multifaceted and not limited to demyelination, cell death and cytotoxic neuroinflammation.

## Conclusion

Our results show that IL-1β has a strong negative effect on axon plasticity, lesion development and gliosis after SCI, associated with substantially impaired functional outcome. In particular, mice with constitutive absence of IL-1β revealed a close-to-opposite effect compared with mice treated with rIL-1β, since IL-1βKO mice were characterized by a smaller lesion size, less astrogliosis and a greater number of labeled CST fibers caudal to the lesion, which resulted in a significantly improved neurological outcome. These data provide a strong basis for further studies to be conducted with the goal of developing therapeutic strategies targeting the IL-1β pathways in spinal cord recovery after injury.

## Abbreviations

BDA: Biotinylated dextran amine; BMS: Basso Mouse Scale; CNS: Central nervous system; CST: Corticospinal tract; GFAP: Glial fibrillary acidic protein; Iba1: Ionized calcium binding adaptor molecule 1; IL: Interleukin; IL-1βKO: IL-1β deficiency; mAb: monoclonal antibody; PBS: Phosphate-buffered saline; rIL-1β: Recombinant IL-1β; SCI: Spinal cord compression injury; WT: Wildtype.

## Competing interests

The authors declare that they have no competing interests.

## Authors’ contributions

FB calculated the mortality rate. FB, KR, SN, LG and SH performed the surgeries, tracing, treated the animals, performed the neurological evaluation and statistics. FB and KR did fibers analysis. FB and EMP did the stainings and analysis of gliosis. SH and FB wrote the manuscript. RN, SH and FB planned the experiments. All authors read and approved the final manuscript.
